# The 2023 revised diagnostic criteria for IgG4-related ophthalmic disease

**DOI:** 10.1007/s10384-024-01072-4

**Published:** 2024-07-22

**Authors:** Masayuki Takahira, Hiroshi Goto, Atsushi Azumi

**Affiliations:** 1 Department of Ophthalmology, Kanazawa University Graduate School of Medical Science, Kanazawa, Japan; 2 Department of Ophthalmology, Tokyo Medical University, Tokyo, Japan; 3 Department of Ophthalmology, Kobe Kaisei Hospital, Hyogo, Japan

**Keywords:** IgG4-related disease, IgG4-related ophthalmic disease, Optic neuropathy, MALT lymphoma, Lacrimal gland

## Abstract

Immunoglobulin G4 (IgG4)-related disease is a clinical entity characterized by elevated serum IgG4 concentrations and infiltration of IgG4-immunopositive plasmacytes in various organs, including ophthalmic lesions. Diagnostic criteria for IgG4-related ophthalmic disease (IgG4-ROD) were established in 2014 and describe the most affected ocular adnexal tissues such as lacrimal glands, trigeminal nerves and extraocular muscles, but do not mention optic neuropathy, the most severe indication of ophthalmic lesions. We reviewed published case reports of optic neuropathy in IgG4-related disease (*n* = 44), and in many cases, decreased visual acuities recovered well following treatment such as systemic corticosteroids, rituximab, and orbital surgery. However, some patients did not recover, especially when pretreatment visual acuities were as low as light perception or less. Herein, we propose a 2023 revised diagnostic criteria for IgG4-ROD, which include a reminder not to overlook optic neuropathy. The 2014 diagnostic criteria specify mucosa-associated lymphoid tissue (MALT) lymphoma as an important differential diagnosis for the relationship between IgG4-ROD and orbital lymphoma. The 2023 revision directs physicians’ attention toward lymphomas other than MALT lymphoma, considering that the 2014 criteria might have placed too much emphasis on MALT lymphoma.

## Introduction

Immunoglobulin G4 (IgG4)-related disease (IgG4-RD) is a clinical entity characterized by IgG4-immunopositive lesions in various body organs accompanied by elevated serum IgG4. Comprehensive diagnostic criteria for IgG4-RD were established in 2011 by the Research Program for Intractable Disease of the Ministry of Health, Labor, and Welfare (MHLW) in Japan [1]. In 2011, the first international symposium of IgG4-RD was held and its conclusions were published as recommendations for the nomenclature [2] and consensus statement on the pathology of IgG4-RD [3]. A representative condition for ophthalmic lesions of IgG4-RD is bilateral IgG4-positive lacrimal gland swelling accompanied by swollen salivary glands, known as IgG4-related Mikulicz’s disease [4]. In 2014, a Japanese ophthalmology study group established diagnostic criteria for IgG4-related ophthalmic disease (IgG4-ROD) [5]. The criteria describe the most affected ocular adnexal tissues of IgG4-ROD: lacrimal glands, trigeminal nerves, and extraocular muscles. However, as clinical knowledge and the literature accumulated, it became apparent that the most severe symptom of ophthalmic lesions is visual loss due to optic neuropathy [6], which we now recommend for the diagnostic criteria for IgG4-ROD. We herein propose the 2023 revised diagnostic criteria for IgG4-ROD.

### Original 2014 diagnostic criteria for IgG4-related ophthalmic disease

The original diagnostic criteria for IgG4-ROD were published in 2014 [5] and comprise three main items: (1) Imaging studies show enlargement of the lacrimal gland, trigeminal nerve, or extraocular muscle as well as masses, enlargement, or hypertrophic lesions in various ophthalmic tissues; (2) Histopathologic examination shows marked lymphocyte and plasmacyte infiltration, and sometimes fibrosis. A germinal center is frequently observed. IgG4 + plasmacytes are found and satisfy the following criteria: ratio of IgG4 + cells to IgG + cells of 40% or above, or more than 50 IgG4 + cells per high-power field (×400); (3) Blood test shows elevated serum IgG4 (> 135 mg/dL) [5]. The first category (1) describes three major lesions of the IgG4-ROD, lacrimal glands, trigeminal nerves, and extraocular muscles, but the criteria do not mention optic neuropathy causing decreased visual acuity and/or visual field impairment.

A recent multicenter study in Japan of 378 patients with IgG4-ROD supports the three most frequent lesions, which are in the lacrimal glands (86%), extraocular muscles (21%), and trigeminal nerves (20%) [6]. The study also describes major ophthalmic symptoms, including dry eye (22%), diplopia (20%), decreased vision (8%), and visual field defects (5%) [6]. The latter two symptoms due to optic neuropathy may lead to blindness. Therefore, we recommend the mention of severe symptoms in the revised version of the diagnostic criteria for IgG4-ROD.

### Optic neuropathy in IgG4-RD

To our knowledge, patients with decreased visual acuity due to optic neuropathy in IgG4-RD were first reported in 2011 [7, 8]. Thereafter, other patients with cases of optic neuropathy caused by IgG4-RD were reported to date [9–37], and are summarized in Table 1. The references in Table 1 were selected using a PubMed search for reports describing case findings including visual acuities published before May 2023, using the keywords, “IgG4 optic neuropathy,” “IgG4 optic perineuritis,” and “IgG4 pachymeningitis visual loss,” and by examining the reference lists of the articles found by the search. The ages of the patients ranged from 17 to 86 (median 61, *n* = 43) years, and the ratio of male-to-female patients was 30/14 (*n* = 44), indicating male dominance. Serum IgG4 levels ranged from 57.9 to 2650 mg/dL (median 355 mg/dL, *n* = 27). Magnetic resonance imaging (MRI) and computed tomography (CT) findings revealed that the most frequent causes of optic neuropathy were compression by a mass around the optic nerve, swollen extraocular muscles, and swollen supraorbital nerve. By contrast, other reports describe optic perineuritis accompanied by lesions of the optic nerve in the orbit and the optic canal, optic chiasma and cavernous sinus, and by hypertrophic pachymeningitis (Table 1, cases :12, 13, 14, 17, 19, 24, 27, 37, 38, and 44). In many cases, decreased visual acuity recovered well through systemic corticosteroids, rituximab, and orbital decompression surgery. Even though the pretreatment visual acuities such as counting fingers or hand motion were very low, visual acuity recovered somewhat after treatment (Table 1, cases: 14, 21, 33, 36, and 44). However, deteriorated visual acuities in other eyes did not recover, (Table 1, cases:13, 14, 24, 29, 30, 32, 37, and 39), especially in patients with severe pretreatment decreased visual acuities, such as light perception or less (cases:13, 14, 37).

**Table 1:** Caption missing

Case #	Report	Reference	Age (years) /gender	Laterality	Pretreatment serum IgG4 (mg/dL)	MRI/CT findings causing optic neuropathy	Pretreatment visual field defect	Pretreatment visual acuity	Posttreatment visual acuity	Treatment
1	Plaza et al. 2011	7	37/M	R	ND	ON sheath mass	ND	ND(decreased)	ND	RTX
2	Higashiyama et al. 2011	8	68/F	L	2170	EOM swelling	ND	0.4	1.5	PS, OS
3	Kubota et al. 2012	9	60/M	R	223	ON sheath/EOM infiltrative lesions	Inferior defect	20/20	ND	ND
4	Sogabe et al. 2014	10	60/F	L	ND	SON swelling compressing ON	ND	0.1	ND	ND
5	Sogabe et al. 2014	10	57/M	L	ND	SON swelling, mass compressing ON	ND	0.4	ND	ND
6	Sogabe et al. 2014	10	36/F	R	ND	Diffuse fat lesion	ND	0.6	ND	ND
7	Sogabe et al. 2014	10	73/M	L	ND	SON swelling, mass compressing ON	ND	0.3	ND	ND
8	Sogabe et al. 2014	10	73/M	L	ND	SON swelling compressing ON	ND	0.6	ND	ND
9	Sogabe et al. 2014	10	58/M	R	ND	Orbital mass compressing ON	ND	0.9	ND	ND
10	Takahashi et al. 2014	11	62/M	R	1850	Mass surrounding ON	Enlarged blind spot	20/20	20/20	PS, OS
				L			Pericecal scotoma	20/125	20/20	
11	Chen et al. 2014	12	74/M	R	382	EOM swelling		NLP	6/36	PS, OS, RTX, RT
12	Tsugawa et al. 2014	13	75/F	R	57.9	Hypertrophic pachymeningitis	ND	HM	1.0	PS, OS
				L			ND	HM	1.2	
13	Lee et al. 2015	14	54/M	R	148	Pachymeningeal lesions with ON shesth	ND	NLP	LP	PS, MTX
				L			ND	NLP	LP	
14	Behbehani et al. 2015	15	36/M	R	144*	Lesions in prechiasmal ON	ND	LP	CF	PS, OS, RTX
				L	*during therapy	Lesions in intracranial ON, chiasm	ND	HM	20/200	
15	Noshiro et al. 2015	16	39/M	R	883	Mass surrounding ON	ND	ND(blindness)	ND	OS, OP
16	Zhang et al. 2016	17	79/F	L	2440	ON atrophy (hyperintense lesion)	ND	0.03	0.3	OS
17	Hwang et al. 2016	18	78/M	R/L	162	Lesions in bilateral cavernous sinuses, optic canal, orbit apex	ND	ND(visual loss)	ND	PS, CPY
18	Wick et al. 2016	19	61/M	R	normal	Mass in the cavernous sinus	ND	20/100	20/50	PS, OS
19	Nakata 2016 (Japanese)	20	70/M	R	355	Lesions surrounding ON, swelling of the cavernous sinus	ND	0.15	0.5	no therapy
20	Wu et al. 2017	21	17/F	R	511	SON swelling compressing ON	Almost total defect	2.0 LogMAR	0 LogMAR	OP, PS, RTX, CPY, OS
21	Takeishi 2017	22	76/F	R	446	EOM swelling, orbital mass compressing ON	Central scotoma	CF	0.6	PS, OS
22	Gorostis 2017	23	61/M	R	191	Orbital mass invading ON	ND	ND	ND	OS, RTX, OP
23	Della-Torre 2018	24	young adult/F	R	ND	Diffuse pachymeningitis	Total defect	ND(blindness)	ND	PS, OS, RTX
				L				ND	ND	
24	Lemaitre 2018	25	78/M	R	71	Optic perineuritis	Normal	20/20	20/50	OS, RTX
				L			Superior scotoma	20/40	CF	
25	Khandji et al. 2018	26	40/M	R	ND	EOM enlargement	Inferior scotoma	20/50	20/20	PS, OS, RTX
26	Yoshinaga et al. 2019	27	74/F	L	98	Lesions in ON/cavernous sinus	Inferior scotoma	0.2	1.2	OS
27	Lai et al. 2020	28	67/M	R	ND	Orbital mass compressing ON	ND	20/70	20/40	PS, OS
28	Lai et al. 2020	28	52/F	R	ND	Orbital mass compressing ON	ND	20/40	20/30	PS, AZA
29	Lai et al. 2020	28	28/F	L	ND	Compressive lesion at cavernous sinus	ND	20/1200	CF	PS, OS, RTX, OP
30	Lai et al. 2020	28	69/M	L	ND	Lobulated infiltration around ON	ND	20/50	20/220	PS, OS, AZA
31	Lai et al. 2020	28	50/F	R	ND	Infiltration around ON	ND	20/30	20/20	PS, OS, AZA, OP
32	Lai et al. 2020	28	19/F	L	ND	Orbital mass compressing ON	ND	20/1000	NLP	PS, OS, OP, infliximab, MTX
33	Oh et al. 2020	29	65/M	R	ND	Mass surrounding ON	ND	CF	20/30	OS
34	Hung et al. 2020	30	38/M	R	2650	Lesions surrounding ON	ND	20/30	20/20	OS, AZA
				L			ND	20/40	20/25	
35	Noda et al. 2021	31	63/M	R	1255	Mass surrounding ON	ND	0.7 LogMAR	-0.1 LogMAR	OP, OS
36	Kim et al. 2021	32	61/M	L	211	Mass surrounding ON	ND	CF	20/40	PS, OS
37	Bae et al. 2021	33	74/M	R	233	Pachymeningitis	ND	NLP	NLP	PS, OS, OP
				L			Central scotoma	6/9	6/9	
38	Woo et al. 2021	34	86/M	R	202	Hypertrophic pachymeningitis	ND	NLP	0.016	PS, OS
				L			ND	0.016	0.1	
39	Detiger et al. 2022	35	19/M	L	281	Sphenoid sinus mucocele comressing ON	ND	20/50	NLP	PS, OS, RTX, OP
40	Hamaoka et al. 2022	36	52/M	R	949	Lesions surrounding ON	Central scotoma	0.8	1.2	OS
				L				0.6	1.2	
41	Hamaoka et al. 2022	36	60/M	L	463	Lesions surrounding ON	ND	0.2	0.4	OS
42	Hamaoka et al. 2022	36	67/M	R	2090	Lesions surrounding ON	Central scotoma	0.2	1.2	OS
				L				0.02	0.04	
43	Hamaoka et al. 2022	36	69/M	L	404	Lesions surrounding ON	Central scotoma	0.6	0.9	OS
44	Balaban et al. 2023	37	68/F	R	128	Lesions in the optic chiasm, orbital apices, cavernous sinuses.	ND	20/40	20/25	RTX
				L			ND	CF	20/60	

Abbreviations: ND: no data; CF: counting fingers; HM: hand motion; LP: light perception; NLP=no light perception

MRI/CT findings: ON: optic nerve; EOM: extraocular muscles; SON: supraorbital nerve

Treatment: PS: pulse steroid; OS: oral steroid; RTX: rituximab; RT: radiation therapy; OP: operation: orbital decompression;

MTX: methotrexate; CPY: cyclophosphamide; AZA: azathioprine

**Fig. 1:**
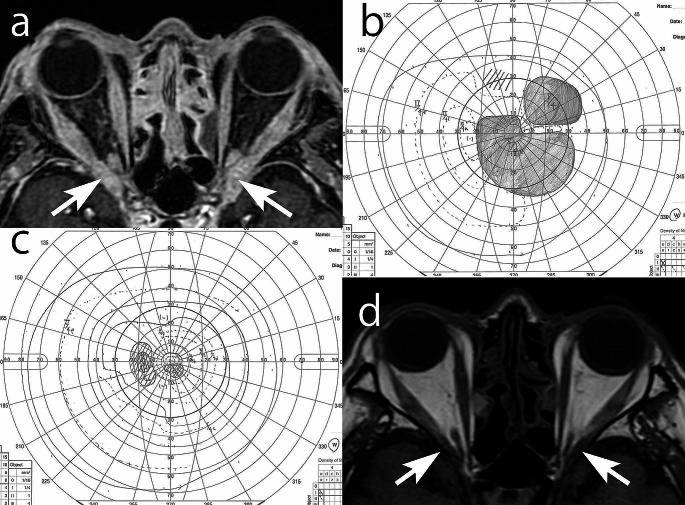
A case of optic neuropathy in IgG4-related disease treated by steroid pulse therapy. **a**) Magnetic resonance imaging (MRI) before therapy shows bilateral extraocular muscle swelling and mass lesions at the orbital apex (arrows) compressing the optic nerves. **b**) After the first course (3 days) of steroid pulse therapy, the left visual field presents central scotoma (visual acuity 0.5). **c**) After the second course of steroid pulse therapy, the left visual field shows only paracentral scotoma (visual acuity 0.8). **d**) MRI after intravenous and oral steroid therapy shows that the orbital apex mass lesions disappeared (arrows)

A representative case of IgG4-ROD with optic neuropathy is presented in Fig. 1, in which visual function responded well to corticosteroid therapy. We obtained the approval of the Kanazawa University Institutional Review Board (IRB)/Ethics Committee to review the case. An elderly man presented with decreased visual acuity to counting fingers OS during the past month. His right best-corrected visual acuity was 0.9 at the initial visit. MRI showed bilateral external ocular muscles swelling and mass lesions at the orbital apex compressing the optic nerves (Fig. 1a). Hematologic studies revealed elevated serum levels of IgG4 746 mg/dL (normal range < 135 mg/dL), suggesting a diagnosis of IgG4-related optic neuropathy. He immediately underwent steroid pulse therapy, and after the first course of intravenous methylprednisolone (1000 mg/day for 3 days), the visual acuities increased to 1.0/0.5 (OD/OS), and the visual field presented paracentral scotoma OD and large central scotoma OS (Fig. 1b). After the second course, the visual acuities increased to 1.2/0.8 (OD/OS), the right visual field improved to normal, and the left visual field showed only paracentral scotoma (Fig. 1c). He underwent three courses of steroid pulse therapy, followed by oral prednisolone tapering therapy with an initial dose of 30 mg/day, resulting in the left visual acuity improving to 1.0. MRI after this steroid therapy showed that the orbital apex mass lesions disappeared (Fig. 1d). This case and previous studies teach us that we should keep in mind the possibility of IgG4-ROD as a differential diagnosis of optic neuropathy and that prompt steroid therapy for IgG4-ROD can lead to a good prognosis.

### IgG4-related ophthalmic disease and lymphoma

Some studies have found that mucosa-associated lymphoid tissue (MALT) lymphoma can arise in the background of IgG4-ROD [38–40]. In addition, a case of IgG4-producing orbital MALT lymphoma de novo is reported [41]. A multicenter study of orbital lymphoproliferative diseases in Japan [42] indicates that MALT lymphoma is an important differential diagnosis of IgG4-ROD. Based on these reports, the original diagnostic criteria of IgG4-ROD in 2014 states that “Mucosa-associated lymphoid tissue (MALT) lymphoma may contain IgG4 + cells; therefore, careful differentiation is necessary” [5]. However, lymphomas other than MALT lymphoma related to IgG4-ROD are also reported: these were follicular lymphoma [38, 43] and diffuse large B cell lymphoma [44–47]. We should be vigilant for chronic inflammatory states of IgG4-ROD, which could cause the development of not only MALT lymphoma but also other B-cell lymphomas.

### The 2023 revised diagnostic criteria for IgG4-related ophthalmic disease

To improve elucidation over the initial version of the diagnostic criteria for IgG4-ROD, a revised version of the diagnostic criteria is proposed for 2023, as shown in Table 2. The revised diagnostic criteria were established through discussions among members of the IgG4-ROD team organized by the Research Program for Intractable Disease by the Ministry of Health, Labor and Welfare (MHLW), Japan. In this revised version, the three main items and the list of differential diagnoses remain unchanged. However, the most severe symptom of IgG4-ROD, visual loss, should be mentioned, and in accordance, a section labeled Attention I was added with the following statements. Clinicians must be vigilant for optic neuropathy causing visual acuity and field deterioration. Attention needs to be paid to hypertrophic pachymeningitis as the cause of optic neuropathy. Another section, Attention II was added, relating to the possibility of IgG4-positive lymphomas other than MALT lymphoma. Lymphomas, as represented by mucosa-associated lymphoid tissue (MALT) lymphoma, may contain IgG4 + cells; therefore, careful differentiation is necessary.

**Table 2:** The 2023 revised diagnostic criteria for IgG4-related ophthalmic disease

(1) Imaging studies show enlargement of the lacrimal gland, trigeminal nerve, or extraocular muscle as well as masses, enlargement, or hypertrophic lesions in various ophthalmic tissues
(2) Histopathologic examination shows marked lymphocyte and plasmacyte infiltration, and sometimes fibrosis. A germinal center is frequently observed. IgG4+ plasmacytes are found and satisfy the following criteria: ratio of IgG4+ cells to IgG+ cells of 40% or above, or more than 50 IgG4+ cells per high-power field (×400). (3) Blood test shows elevated serum IgG4 (>135 mg/dl) Diagnosis is classified as ‘‘definitive’’ when (1), (2), and (3) are satisfied; “probable’’ when (1) and (2) are satisfied; and ‘‘possible’’ when (1) and (3) are satisfied Differential diagnosis of IgG4-related ophthalmic disease Sjögren syndrome, Lymphoma, Sarcoidosis, Granulomatosis with polyangiitis (Wegener granulomatosis), Thyroid-related orbitopathy, Idiopathic orbital inflammation, Dacryoadenitis or orbital cellulitis caused by bacteria or fungi Attention I) Clinicians must be vigilant for optic neuropathy, causing visual acuity and field deterioration. Attention needs to be paid to hypertrophic pachymeningitis as the cause of optic neuropathy. II) Lymphomas, as represented by mucosa-associated lymphoid tissue (MALT) lymphoma, may contain IgG4+ cells; therefore, careful differentiation is necessary.

### Relationship to the comprehensive diagnostic criteria for IgG4-related disease

The initial version of the “comprehensive diagnostic criteria for IgG4-related disease” was established in 2011 [1], and is used to diagnose IgG4-RD in all organs. These comprehensive diagnostic criteria were revised in 2020 [48]. In the 2020 revised comprehensive diagnostic (RCD) criteria [48], Item 3, pathological diagnosis is described as: Positivity for two of the following three criteria, (1) Dense lymphocytes and plasma cell infiltration with fibrosis, (2) Ratio of IgG4-positive plasma cells /IgG-positive cells greater than 40% and the number of IgG4-positive plasma cells greater than 10 per high powered field. (3) Typical tissue fibrosis, particularly storiform fibrosis, or obliterative phlebitis.

We found that although some cases proved definite according to the diagnostic criteria of IgG4-ROD, they did not meet the definite 2020 RCD criteria [48] because fibrosis was not always seen in biopsy samples. For instance, in a 38-case series according to the diagnostic criteria for IgG4-ROD at Kanazawa University Hospital, 5 cases failed to meet the 2020 RCD criteria because there was no fibrosis in the biopsy samples (unpublished observation). However, an Explanatory note 1 in the 2020 RCD criteria describes the combination of organ-specific diagnostic criteria as, patients with a possible or probable diagnosis by comprehensive diagnostic criteria who fulfill the organ-specific criteria for IgG4-RD are regarded as being definite for IgG4-RD [48]. Therefore, patients with IgG4-ROD without fibrosis can also be considered definite.

### Perspective

The main purpose of the present article is to establish optic neuropathy as the most severe symptom of IgG4-ROD in the diagnostic criteria. Another severe symptom of IgG4-ROD is diplopia, ocular movement impairment caused by extraocular muscle swelling and/or orbital mass lesions. The effect of systemic corticosteroids for these symptoms of IgG4-ROD is generally good, but in some cases there are limitations in its recovery. Therefore, we highly recommend prompt adoption of corticosteroid therapy.

IgG4-RD has been designated as a specified intractable disease (specific disease) in Japan, but the criteria does not include optic neuropathy. A treatment guideline for IgG4-RD describing serious circumstances, including optic neuropathy, is warranted.

## Appendix

The members of the Japanese Study Group for IgG4-Related Ophthalmic Disease organized by the Research Program for Intractable Disease by the Ministry of Health, Labor and Welfare of Japan are as follows:

Atsushi Azumi, Department of Ophthalmology, Kobe Kaisei Hospital.

Minoru Furuta. Department of Ophthalmology, Fukushima Medical University / Soma Central Hospital.

Hiroshi Goto, Department of Ophthalmology, Tokyo Medical University.

Kazuko Kitagawa, Department of Ophthalmology, Kanazawa Medical University.

Yoko Ogawa, Shinjuku City Eye Clinic / Department of Ophthalmology, Keio University School of Medicine.

Koh-ichi Ohshima, Department of Ophthalmology, National Hospital Organization Okayama Medical Center.

Tokuhide Oyama, Medical Corporation Oculus, Uonuma eye clinic, Niigata / Division of Ophthalmology and Visual Sciences, Graduate School of Medical and Dental Sciences, Niigata University.

Yuka Sogabe, Department of Ophthalmology, Mitoyo General Hospital.

Shigenobu Suzuki, Department of Ophthalmic Oncology, National Cancer Center Hospital.

Masayuki Takahira, Department of Ophthalmology, Kanazawa University Graduate School of Medical Science.

Hideki Tsuji, Department of Ophthalmology, The Cancer Institute Hospital of the Japanese Foundation of Cancer Research.

Yoshihiko Usui. Department of Ophthalmology, Tokyo Medical University.
